# Theaflavin-3,3′-Digallate Suppresses Human Ovarian Carcinoma OVCAR-3 Cells by Regulating the Checkpoint Kinase 2 and p27 kip1 Pathways

**DOI:** 10.3390/molecules24040673

**Published:** 2019-02-14

**Authors:** Ying Gao, Junfeng Yin, Youying Tu, Yi Charlie Chen

**Affiliations:** 1 Tea Research Institute Chinese Academy of Agricultural Sciences, Ministry of Agriculture, Hangzhou 310008, China; yinggao@tricaas.com (Y.G.); yinjf@tricaas.com (J.Y.); 2 Department of Tea Science, Zhejiang University, Hangzhou 310058, China; 3 College of Science, Technology and Mathematics, Alderson Broaddus University, Philippi, WV 26416, USA

**Keywords:** theaflavin-3,3′-digallate, apoptosis, death receptors, Chk2, cell cycle arrest, p27

## Abstract

Theaflavin-3,3′-digallate (TF3) is a unique polyphenol in black tea. Epidemiological studies have proved that black tea consumption decreases the incidence rate of ovarian cancer. Our former research demonstrated that TF3 inhibited human ovarian cancer cells. Nevertheless, the roles of checkpoint kinase 2 (Chk2) and p27 kip1 (p27) in TF3-mediated inhibition of human ovarian cancer cells have not yet been investigated. In the current study, TF3 enhanced the phosphorylation of Chk2 to modulate the ratio of pro/anti-apoptotic Bcl-2 family proteins to initiate intrinsic apoptosis in a p53-independent manner and increased the expression of death receptors to activate extrinsic apoptosis in OVCAR-3 human ovarian carcinoma cells. In addition, TF3 up-regulated the expression of p27 to induce G0/G1 cell cycle arrest in OVCAR-3 cells. Our study indicated that Chk2 and p27 were vital anticancer targets of TF3 and provided more evidence that TF3 might be a potent agent to be applied as adjuvant treatment for ovarian cancer.

## 1. Introduction

Ovarian cancer is the fifth most deadly cancer in women—an estimated 22,240 new cases of ovarian cancer are expected in the United States in 2018, with an estimated 14,070 deaths [1]. Despite the improvement of medical diagnosis and treatment, the prognosis of ovarian cancer remains poor. Patients are at high risk of having a relapse of the disease within five years [2], and recurrent ovarian cancer is typically resistant to chemotherapeutic strategies [2]. Besides, current treatment often causes a range of side-effects [3]. Thus, it is necessary to develop novel cancer treatments. Polyphenols, a class of chemicals characterized by the presence of large multiples of phenol structural units, are regarded as potential anticancer candidates because they have multiple molecular targets, high efficiency and little adverse side effects [4].

**Figure 1:**
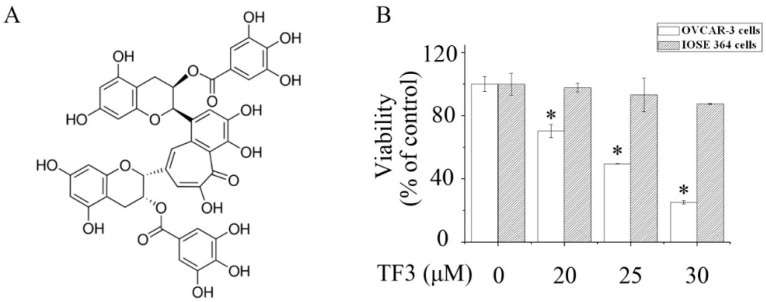
Theaflavin-3,3′-digallate (TF3) selectively reduced the viability of ovarian cancer cells. (**A**) Chemical structure of TF3. (**B**) The viability of human ovarian carcinoma (OVCAR-3) cells and normal human immortalized ovarian surface epithelial cells (IOSE 364) cells exposed to TF3. * *p* < 0.05 compared with the control group.

Theaflavin-3,3′-digallate (TF3) (Figure 1A) is a unique black tea polyphenol, which is produced by oxidation and dimerization of (−)-epigallocatechin-3-gallate (EGCG) and epicatechin gallate during the fermentation process. Epidemiological studies have shown that black tea consumption reduces ovarian cancer risk [5]. Our previous research elucidated that TF3 suppressed tumor angiogenesis by inhibiting the Akt and Notch-1 pathways in human ovarian cancer cells [6]. However, whether TF3 can inhibit ovarian cancer via other pathways remains unclear. In this study, the effects of TF3 on apoptosis, as well as cell cycle distribution, were assessed and whether checkpoint kinase 2 (Chk2) and p27 kip1 (p27) were involved in TF3-mediated inhibition of human ovarian cancer cells was also investigated.

## 2. Results and Discussion

### 2.1. TF3 Preferentially Decreases the Viability of Human Ovarian Carcinoma (OVCAR-3) Cells

As shown in Figure 1B, exposure to TF3 remarkably reduced the viability of OVCAR-3 cells in a concentration-dependent manner. Meanwhile, TF3 hardly affected normal human immortalized ovarian surface epithelial cells (IOSE 364). This indicated that the cytotoxicity of TF3 was selective to ovarian cancer cells.

### 2.2. TF3 Activates Apoptosis in OVCAR-3 Cells

The stimulation of apoptosis is a mechanism shared by most chemotherapeutic agents. Apoptosis describes the orchestrated collapse of a cell characterized by membrane blebbing, cell shrinkage, condensation of chromatin, and fragmentation of DNA followed by rapid engulfment of the corpse by neighboring cells [7]. Unlike necrosis, apoptosis does not cause inflammation. Apoptosis can be triggered via the intrinsic and extrinsic pathways—the intrinsic pathway is initiated by stress signals, followed by the increased permeability of the mitochondria and the release of apoptogenic factors (e.g., cytochrome c) into the cytosol. Subsequently, cytochrome c and Apaf-1 form the apoptosome, which recruits and activates pro-caspase-9 triggering the cleavage of effector caspases (e.g., Caspase-3 and Caspase-7) and finally apoptosis [8]. The extrinsic pathway is stimulated by the binding of death receptors with their corresponding ligands. This results in receptor aggregation and recruitment of the adaptor molecules, which in turn recruit Caspase-8, forming the death-inducing signaling complex (DISC) [8]. Oligomerization of Caspase-8 upon DISC formation drives its activation through self-cleavage [8]. Caspase-8 then activates downstream effector caspases, which are able to cleave down-stream proteins (e.g., poly [ADP-ribose] polymerase 1 (PARP-1)). Cleavage of PARP-1 by caspases is considered to be a hallmark of apoptosis [9].

**Figure 2:**
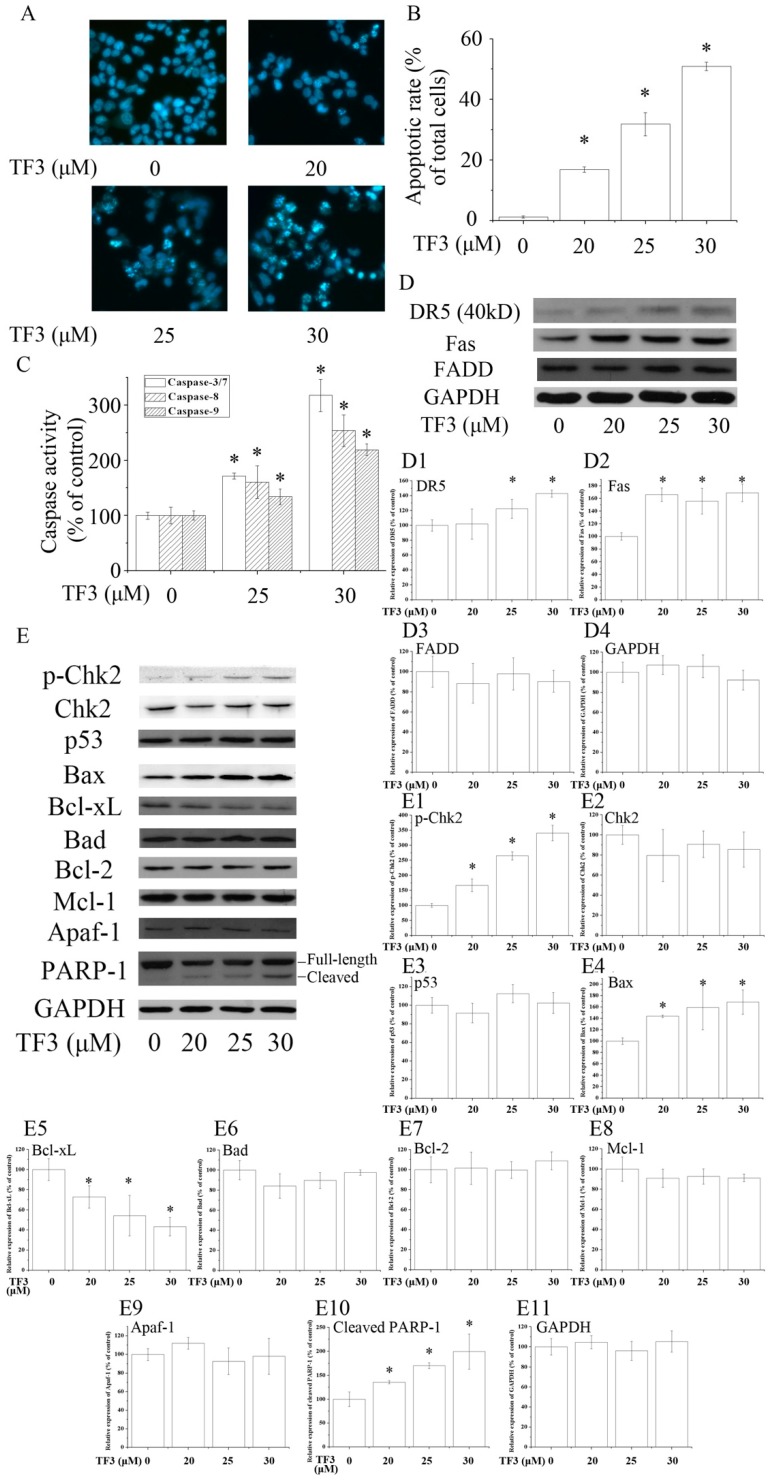
Theaflavin-3,3′-digallate (TF3) induced apoptosis in human ovarian carcinoma (OVCAR-3) cells. (**A**,**B**) Hoechst 33342 staining assay. (**C**) TF3 activated caspases in OVCAR-3 cells. (**D**,**E**) TF3 influenced apoptosis-related proteins. (**D1**–**D4** & **E1**–**E11**) Densitometry analysis of protein bands. Glyceraldehyde-3-phosphate dehydrogenase (GAPDH) served as a loading control. * *p* < 0.05 compared with the control group.

To evaluate whether TF3 induced apoptosis in OVCAR-3 cells, Hoechst 33342 staining assay was carried out. Based on the results, TF3 dose-dependently increased the apoptotic rate of OVCAR-3 cells (Figure 2A,B). In accordance with the result of Hoechst 33342 staining assay, TF3 markedly increased the level of cleaved PARP-1 and activated Caspase-3/7, 8 and 9, respectively (Figure 2C,E). This hinted that TF3 triggered intrinsic and extrinsic apoptosis.

### 2.3. TF3 Mediates Aoptosis through Death Receptors and Chk2

Death receptors are involved in the initiation of the extrinsic apoptotic pathways. Death receptor 5 (DR5) and Fas are two key members of the death receptor family and are the targets of many anticancer agents. The up-regulation of death receptors sensitizes cancer cells to apoptosis. In the current study, TF3 dramatically increased the protein levels of DR5 and Fas (Figure 2D). The expression of Fas-Associated protein with Death Domain (FADD), an adaptor protein that bridges members of the tumor necrosis factor receptor superfamily, was not influenced. The above results implicated that TF3 mainly triggered extrinsic apoptosis through the up-regulation of death receptors. Former research elucidated that theaflavins and EGCG targeted Fas/Caspasae-8 triggered apoptosis [10,11]. Our results supplied new evidence that the death receptors/Caspase-8 pathway participated in TF3-induced apoptosis.

The Bcl-2 family is most notable for the regulation of intrinsic apoptosis by governing mitochondrial outer membrane permeabilization, a vital step in the intrinsic apoptosis. The Bcl-2 family consists of two members which display opposite functions—the anti-apoptotic Bcl-2 proteins (e.g., Bcl-2, Bcl-xL and Mcl-1) prevent apoptosis, either by sequestering pro-caspases or by inhibiting the release of mitochondrial apoptogenic factors into the cytosol [12]. In contrast, pro-apoptotic Bcl-2 proteins, such as Bax and Bad, promote the release of caspases and mitochondrial apoptogenic factors, thereby leading to caspase activation [12]. The ratio of pro to anti-apoptotic Bcl-2 proteins determines the cell fate. In this case, we found TF3 up-regulated the level of Bax and down-regulated the level of Bcl-xL, leading to an increased pro to anti-apoptotic Bcl-2 protein ratio. Nevertheless, it did not change the expressions of Bad, Bcl-2 and Mcl-1 (Figure 2E).

P53 is a key tumor suppressor which plays multiple roles in anticancer. P53 promotes intrinsic apoptosis by modulation of the Bcl-2 family proteins. Our former work showed that TF3 induced apoptosis via targeting the p53 and the Bcl-2 family proteins in p53-wild-type ovarian cancer cells [13]. However, loss and/or mutation of p53 are very common in human cancers [14,15]. Induction of p53-independent apoptosis is regarded as a promising way to overcome resistance. Interestingly, in this study, we found the expression of p53 was not influenced by TF3, which implied that TF3 activated apoptosis independently of p53 in p53-mutant OVCAR-3 cells. Our results suggested that p53 might not be a prerequisite for TF3-induced apoptosis and TF3 had the potential to be applied in cancer treatment for sensitizing p53-mutant cancer cells to apoptosis.

**Figure 3:**
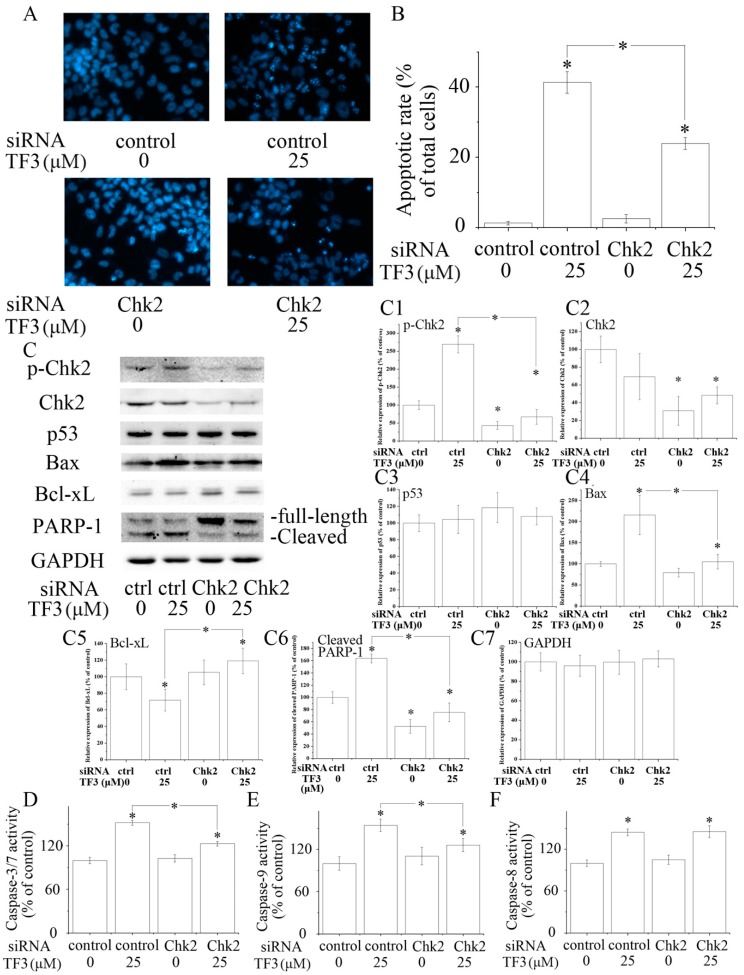
The role of checkpoint kinase 2 (Chk2) in theaflavin-3, 3′-digallate (TF3)-induced apoptosis. (**A**,**B**) Blocking Chk2 impaired TF3-induced apoptosis. (**C**) Blocking Chk2 partially reversed TF3-induced increase of the expression of p-Chk2, Bax and cleaved poly (ADP-ribose) polymerase 1 (PARP-1), as well as a decrease of the expression of Bcl-xL. (**C1**–**C7**) Densitometry analysis of protein bands. (**D**–**F**) Blocking Chk2 attenuated the activation of Caspase-3/7 and 9 induced by TF3. However, the activity of Caspase-8 was not affected. * *p* < 0.05 compared with control or between specific groups.

Chk2 acts as a key signal transducer of cellular responses and a candidate tumor suppressor [16]. Once activated, Chk2 can induce apoptosis with or without the presence of p53 [17,18]. Mutations and/or deletions of Chk2 are associated with various types of cancers [19]. In this research, TF3 was detected to significantly increase the phosphorylation of Chk2 (Figure 2E). The blocking of Chk2 obviously attenuated TF3-mediated apoptosis and increase of the protein levels of p-Chk2, Bax and cleaved PARP-1, as well as the activation of Caspase-3/7 and Caspase-9 (Figure 3). TF3-mediated reduction of the expression of Bcl-xL was less in Chk2 siRNA transfected cells than in control siRNA transfected cells (Figure 3C). Nevertheless, TF3-mediated Caspase-8 activation was not significantly affected by the knockdown of Chk2 (Figure 3F). This is in accordance with a former study which reported that the activation of Chk2 led to activation of Caspase-9 and subsequently the induction of intrinsic apoptosis [20]. Taken together, these results confirmed that Chk2 was involved in TF3-stimulated intrinsic, but not extrinsic, apoptosis in OVCAR-3 cells.

### 2.4. TF3 Induces G0/G1 Cell Cycle Arrest in OVCAR-3 Cells via p27

Cancer is a disease of inappropriate cell proliferation, which is controlled by cell cycle machinery. Halting the cell cycle not only lowers the rate of proliferation, but also causes apoptosis if the cell cycle is paused too long. Many classic chemotherapeutic agents exert their anticancer properties by disturbing the cell cycle. In this study, the cell cycle distribution of TF3-treated OVCAR-3 cells was determined using the flow cytometry. The results showed that the population of cells in the G0/G1 phase was increased and the population of cells in the S phase was declined after exposure to 20 μM TF3 for 24 h, meaning TF3 caused G0/G1 cell cycle arrest in OVCAR-3 cells (Table 1).

P27 is a cyclin dependent kinase (CDK) inhibitor which negatively regulates CDK activity at the G0/G1 phase transition. The induction of p27 causes G0/G1 cell cycle arrest [21,22]. The loss of expression or function of p27 has been implicated in the genesis or progression of many human malignancies [23]. P27 inhibits the activity of Cyclin D1-CDK4 more effectively than that of Cyclin E-CDK2 [24]. Both Cyclin D1-CDK4 and Cyclin E-CDK2 complexes can phosphorylate Rb. Rb regulates the activity of the transcription factors of the E2F family. Under non-dividing conditions, Rb is hypo-phosphorylated (active) and binds with E2F. Upon hyper-phosphorylation (inactive form), the affinity between Rb and E2F attenuates. E2F is released from Rb and activates the expression of proteins required in the next stages of the cell cycle and in DNA replication (e.g., Cyclin E and Cyclin A) [25].

**Figure 4:**
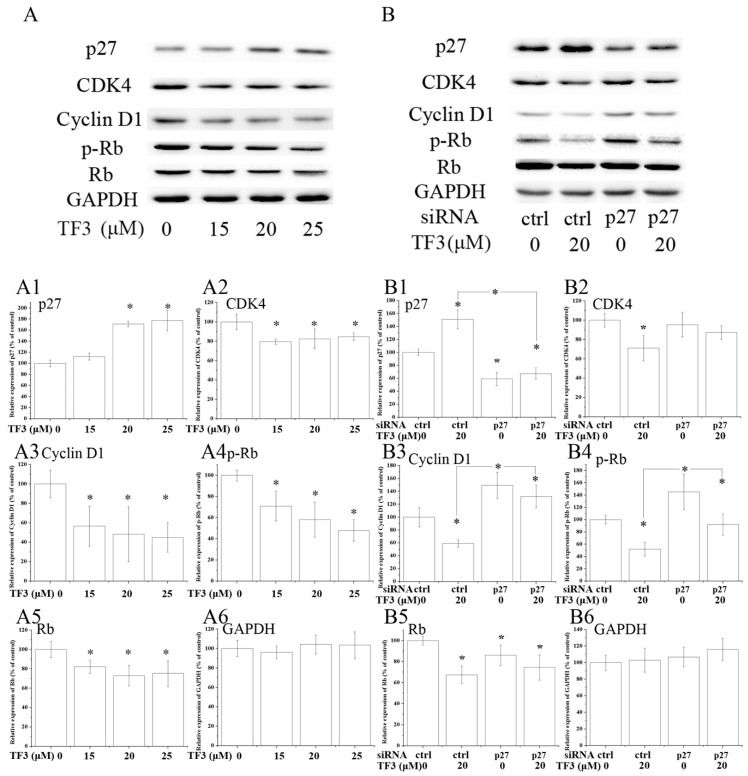
TF3 caused G0/G1 cell cycle arrest in OVCAR-3 cells by up-regulating p27 kip1 (p27). (**A**) Western blot analysis of TF3-treated OVCAR-3 cells. (**B**) Knockdown of p27 partially reversed TF3-induced decrease of the expression of cyclin dependent kinase (CDK)4, Cyclin D1, phospho-retinoblastoma protein (p-Rb) and retinoblastoma protein (Rb) in OVCAR-3 cells. (**A1**–**A6** & **B1**–**B6**) Densitometry analysis of protein bands. Glyceraldehyde-3-phosphate dehydrogenase (GAPDH) served as the loading control. * *p* < 0.05 compared with control or between specific groups.

Western blot analysis illustrated that the expression of CDK4, Cyclin D1, phospho-retinoblastoma protein (p-Rb) and Rb were dramatically decreased in TF3-exposed OVCAR-3 cells (Figure 4A). Meanwhile, the expression of p27 was obviously elevated by TF3 (Figure 4A). The blocking of p27 impaired the effects of TF3 (Table 2) (Figure 4B). In p27 siRNA transfected cells, TF3-mediated up-regulation of p27 and down-regulation of Cyclin D1 and p-Rb were partially reversed compared with that in the control siRNA transfected cells. This data demonstrated TF3 induced G0/G1 cell cycle arrest via the p27/Cyclin D1-CDK4/Rb pathway in OVCAR-3 cells. Similar results have been reported previously in other cancer cell lines. In that study, researchers found theaflavins and thearubigins induced cell cycle arrest at the G0/G1 phase in human leukemic U937 and K562 cells through augmented expression of p19, p21 and p27, while ablating CDK2, CDK4, CDK6 and Cyclin D1 [26]. It hinted that targeting p27 and its down-stream proteins might be a universal strategy of theaflavins to regulate the cell cycle of cancer cells.

## 3. Materials and Methods

### 3.1. Cells and Reagents

Human immortalized ovarian surface epithelial cells (IOSE 364) and human ovarian carcinoma cells (OVCAR-3) were donated by Dr. Auersperg (University of British Columbia, Vancouver, BC, Canada) and Dr. Jiang (Thomas Jefferson University, Philadelphia, PA, USA), respectively. Both cells were grown in RPMI 1640 medium containing 10% fetal bovine serum.

Reagents: TF3 monomer (purity of 92.4%) was prepared by a previously established method [27]. BisBenzimide H 33342 trihydrochloride (Hoechst 33342) were purchased from Sigma (St. Louis, MO, USA). Propidium iodide (PI) was purchased from Invitrogen (Carlsbad, CA, USA). Antibodies against phospho-Chk2 (Thr68), Chk2, p53, Bad, Bcl-xL, PARP-1 and GAPDH were obtained from Santa Cruz Biotechnology Inc. (Santa Cruz, CA, USA). Antibodies against Bax, Bcl-2, Mcl-1, Apaf-1, Fas, DR5, FADD, p27, CDK4, Cyclin D1, p-Rb and Rb were purchased from Cell Signaling Technology, Inc. (Danvers, MA, USA).

### 3.2. Determination of Cell Viability

Cells were seeded into 96-well plates (10^4^/well), incubated overnight (5% CO_2_, 37 °C), exposed to TF3 for 24 h, and then the viability was assessed by a Cell Proliferation Assay kit (CellTiter 96^®^, Promega, Madison, WI, USA). The results were presented as a percentage compared to the control group.

### 3.3. Hoechst 33342 Staining Assay

Cells exposed to TF3 were collected after 24 h treatment, washed with PBS twice, and then stained with Hoechst 33342 (10 µg/mL) for 10 min. Apoptosis was observed using a fluorescence microscope (ZEISS). Cells with condensed or fragmented nuclei were considered as apoptotic cells. Apoptotic rate (%) = (Apoptotic cell number)/(total cell number) × 100.

### 3.4. Determination of Caspase Activities

Cells were exposed to TF3 for 24 h and then the activities of Caspase-3/7, Caspase-8, and Caspase-9 were measured using Caspase-Glo Assay kits (Promega, USA). The results were presented as a percentage compared to the control group.

### 3.5. Determination of Cell Cycle Distribution

Cells were collected after exposure to TF3 for 24 h, fixed with 70% ethanol at −20 °C for 16 h, treated with RNase to remove RNA, stained with PI and then detected via flow cytometry (FACS Calibur, BD Bioscience, San Diego, CA, USA).

### 3.6. Western Blot

Cells were collected after exposure to TF3 for 24 h and lysed to extract proteins. The Western blot assay was carried out based on a published method [6].

### 3.7. Transfection with Small Interfering RNA (siRNA)

Cells were transfected with siRNA using a commercial siRNA transfection reagent (jetPRIME™, VWR International, Radnor, PA, USA) for 24 h following which the cells were exposed to TF3 for 24 h for further tests.

### 3.8. Statistical Analysis

Data were presented as mean ± standard error of mean (SEM) from at least three independent experiments. One-way ANOVA followed by post-hoc test was applied for statistical analysis using SPSS software (IBM, Version 22.0, Armonk, NY, USA). The difference was deemed significant if the *p*-value was less than 0.05.

## 4. Conclusions

**Figure 5:**
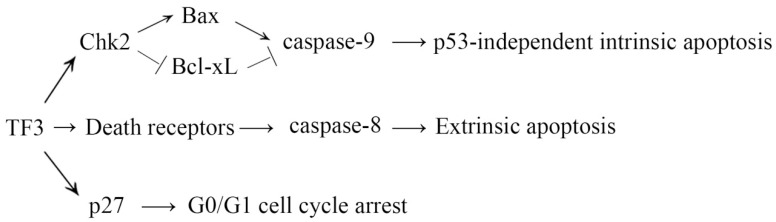
Proposed mechanism for the inhibition of TF3 in OVCAR-3 cells.

In conclusion, TF3 was selectively toxic to ovarian cancer cells rather than normal ovarian cells, indicating TF3 might be a promising anticancer agent candidate with few adverse side effects. TF3 activated extrinsic apoptosis via up-regulation of death receptors and induced intrinsic apoptosis via the Chk2 pathway independently of p53 (Figure 5). TF3 also caused G0/G1 cell cycle arrest via the p27/Cyclin D1-CDK4/Rb pathway (Figure 5). Considering OVCAR-3 cells are cisplatin-resistant and p53-mutant, the current results implicated that TF3 might have the potential to be applied in the treatment of cisplatin-resistant and p53-mutant ovarian cancer. Further studies are needed to assess the efficacy of TF3 in ovarian cancer treatment.

## Figures and Tables

**Table 1:** Cell cycle distribution of TF3-treated OVCAR-3 cells.

TF3 (μM)	Cell Cycle Distribution (%)		
	G0/G1 Phase	S Phase	G2/M Phase
0	60.48 ± 1.34	33.41 ± 0.72	6.12 ± 0.63
10	62.78 ± 1.14	31.42 ± 0.29	5.81 ± 1.43
15	65.12 ± 3.79	29.37 ± 4.37	5.52 ± 0.59
20	69.93 ± 2.68 *	23.65 ± 1.99 *	5.93 ± 1.37

* *p* < 0.05 compared with control or between specific groups.

**Table 2:** Knockdown of p27 partially abrogated TF3-induced G0/G1 cell cycle arrest.

siRNA	TF3 (μM)	Cell Cycle Distribution (%)		
		G0/G1 Phase	S Phase	G2/M Phase
Control	0	62.37 ± 2.66	31.78 ± 1.13	6.01 ± 0.82
Control	20	72.01 ± 3.42 *	22.35 ± 2.16 *	5.77 ± 0.60
p27	0	58.77 ± 2.21	34.07 ± 2.43	7.23 ± 1.79
p27	20	61.66 ± 3.59	33.84 ± 2.78	6.04 ± 1.41

* *p* < 0.05 compared with control or between specific groups.
